# Early Maternal and Social Deprivation Expands Neural Stem Cell Population Size and Reduces Hippocampus/Amygdala-Dependent Fear Memory

**DOI:** 10.3389/fnins.2020.00022

**Published:** 2020-01-29

**Authors:** Kenny Anak Daun, Takahiro Fuchigami, Natsu Koyama, Noriko Maruta, Kazuhiro Ikenaka, Seiji Hitoshi

**Affiliations:** ^1^Department of Integrative Physiology, Shiga University of Medical Science, Otsu, Japan; ^2^Department of Psychiatry, Health Center, Hitotsubashi University, Tokyo, Japan; ^3^Division of Neurobiology and Bioinformatics, National Institute for Physiological Sciences, Okazaki, Japan; ^4^Department of Physiological Sciences, School of Life Sciences, The Graduate University for Advanced Studies, Okazaki, Japan

**Keywords:** stress, amygdala, neural stem cell, corticosterone, fear memory, resilience

## Abstract

Early life stress can exert detrimental or beneficial effects on neural development and postnatal behavior depending on the timing, duration, strength, and ability to control the stressors. In this study, we utilized a maternal and social deprivation (MSD) model to investigate the effects of early life stress on neural stem cells (NSCs) and neurogenesis in the adult brain. We found that MSD during the stress-hyporesponsive period (SHRP) (early-MSD), when corticosterone secretion is suppressed, increased the size of the NSC population, whereas the same stress beyond the SHRP abrogated these effects. Early-MSD enhanced neurogenesis not only in the dentate gyrus of the hippocampus, one of the classic neurogenic regions, but also in the amygdala. In addition, mice exposed to early-MSD exhibited a reduction in amygdala/hippocampus-dependent fear memory. These results suggest that animals exposed to early life stress during the SHRP have reinforced stress resilience to cope with perceived stressors to maintain a normal homeostatic state.

## Introduction

Environmental manipulation during postnatal life predisposes the mammalian brain to altered function and cellular remodeling resulting in behavioral and mood changes. One of the cellular mechanisms contributing to these alterations is adult neurogenesis, which occurs in two neurogenic regions, the olfactory bulb and the dentate gyrus of the hippocampus, in the normal rodent brain (Alvarez-Buylla and Garcıa-Verdugo, 2002; Ming and Song, 2011). Neural stem cells (NSCs) reside in the subependymal zone (SEZ) around the lateral ventricles, recently also referred to as the ventricular–subventricular zone (V-SVZ) based on the spatial arrangement and cellular morphology of NSCs (Lim and Alvarez-Buylla, 2016), and provide new neurons to the olfactory bulb through the rostral migratory stream; they are also present in the subgranular zone (SGZ) where they produce granular cells in the dentate gyrus (Gage, 2000; Seaberg and van der Kooy, 2002). In the neurogenic regions, neurogenesis plays a critical role in the plasticity of brain functions related to these areas, such as olfactory discrimination, memory formation, and extinction (Shors et al., 2001; Alonso et al., 2006; Pan et al., 2012). Recently, it has been reported that adult neurogenesis also occurs in other brain regions, including the hypothalamus and amygdala (Fowler et al., 2008; Lieberwirth et al., 2016; Jhaveri et al., 2018).

The size of the neural precursor population and the extent of adult neurogenesis are under the influence of many external stimuli (Kempermann, 2012). For example, an enriched environment in a rodent’s housing cage, physical exercise, and learning can increase the generation, survival, and maturation of new neurons that are subsequently incorporated into existing neural networks (van Praag et al., 1999; Barha et al., 2011; Lee et al., 2016). Conversely, psychosocial and physical stressors shrink the neural precursor population and attenuate adult neurogenesis (Duman et al., 2001; Malberg, 2004; Mirescu et al., 2004). The hypothalamus–pituitary–adrenal (HPA) axis is one of the critical mediators of these effects as corticosterone directly suppresses the self-renewal of NSCs in the adult brain (Hitoshi et al., 2007). However, relatively little is known about the effects of early life stress, which may be associated with an increased risk for psychosocial difficulties later in life in humans. Indeed, the postnatal hippocampus and amygdala are the most active regions for the neurodevelopment including serial neurogenesis, cell differentiation, and migration, particularly during the first 2 weeks of life. During these periods in rodents, the HPA axis is insensitive to environmental stimuli and this stress-hyporesponsive period (SHRP) is thought to be neuroprotective against the stress-induced excessive release of corticosterone during early postnatal development (Levine, 2002; Schmidt et al., 2003; Fodor and Zelena, 2014; Macrì, 2017). A recent finding showed that early life stress induced by limited bedding and nesting paradigm delayed the dentate gyrus development and depletes adult stem cell pool without affecting neurogenesis (Youssef et al., 2019). However, it remains unclear whether early life stress alters the size of the neural precursor population in SEZ and the adult neurogenesis in brain regions that can predict stress–resilience or vulnerability related to emotion and mood, in addition to the classic neurogenic regions.

Maternal separation (MS) is a well-characterized rodent model for early life stress but shows significant dissimilarities in its effects between studies; this could be due to differences in the start or end of its timing, duration of the separation episodes, and strength of the stressors (Suri et al., 2013; Nishi et al., 2014). For instance, the standard MS (which isolates the dam in a novel cage while the pups are separated together) exaggerated HPA-axis activity in adulthood, a brief separation (referred to as early “handling”) or separation, in which pups are isolated individually while the dam is left with a subset of littermates in the home cage (referred to as “maternal and social deprivation”: MSD), decreased the HPA-axis responses to stress (Caldji et al., 2000; Liu et al., 2000; Kawakami et al., 2007; Sandi and Haller, 2015). In addition, behavioral phenotypes themselves may change depending on the age of the animal: rats that receive early life stress during the SHRP show stronger cued fear memory in young adulthood (8 weeks) but not in adulthood (15 weeks) (Mishra et al., 2019). In this study, we focused on MSD during the SHRP and investigated how the altered NSC population size and adult neurogenesis by this early life MSD affects the emotional behavior and stress response in adulthood.

## Materials and Methods

### Animals

The Animal Care and Use Committee of the Shiga University of Medical Science, in compliance with the guidelines of the National Institutes of Health, approved the use and care of animals in this study. Pregnant C57BL/6J mice were provided on gestational day16 from Japan SLC, Inc. (Hamamatsu, Japan) and their pups with total number of 224 were used in this study. We used only male mice for the behavioral studies and both male and female mice for other experiments. All animals were maintained at room temperature on a 12:12 h light–dark cycle (8 am–8 pm).

### Maternal and Social Deprivation

The day pups were delivered was defined as postnatal day (P) 0. The pups were randomly assigned to one of the following experimental groups: (a) separation from the dam and littermates from P1 to P14 during the SHRP (early-MSD); (b) separation from P15–P21 after the SHRP (late-MSD); and (c) without separation. For the neurosphere and corticosterone assay experiment, an extended-MSD (P1–P21) group was added. MSD and littermate control pups were bred in the same dams. The MSD pups were separated from their dam and littermates for 3 h (1–4 pm). They were placed into paper cups with 8 cm bottom-diameter and 20 cm height, lined with wood-chip bedding. To avoid disturbing the dam by the vocalization of the MSD pups, the cages containing the pups’ cups were moved to a different room from their home cage. The non-separated control mice were undisturbed from their dam except for routine cage changes. On P28, all pups were weaned and group-housed with littermates of the same sex.

### Neural Precursor Cell Culture

The isolation of neural stem cells (NSCs) from adult brains was performed as previously described (Reynolds and Weiss, 1992; Higashi et al., 2008). Briefly, brains of MSD mice were aseptically collected into oxygenized artificial cerebrospinal fluid (124 mM NaCl, 5 mM KCI, 1.3 mM MgCl_2_, 2 mM CaCl_2_, 26 mM NaHCO_3_, 0.18% glucose, and 100 unit/ml penicillin/streptomycin) and the entire SEZ of the lateral ventricles was excised and cut into small pieces. The tissue was subjected to enzyme digestion in a solution containing 1.33 mg/ml trypsin, 0.67 mg/ml hyaluronidase, and 0.2 mg/ml kynurenic acid (all from Sigma) for 1 h at 37°C to facilitate dissociation of the tissue. The tissue was then triturated using a fire-polished Pasteur pipette in serum-free media [SFM; DMEM/F12 (1:1), 5 mM HEPES buffer, 0.6% glucose, 3 mM NaHCO_3_, 2 mM glutamine, 25 μg/ml insulin, 100 μg/ml transferrin, 20 nM progesterone, 60 μM putrescine, and 30 nM sodium selenite (all from Sigma)] containing trypsin inhibitor (Roche). After washing with SFM, the cells were cultured at a density of 5 cells/μl in a 24-well plate (Falcon) in 500 μl of SFM containing 20 ng/ml EGF, 10 ng/ml FGF2, and 2 μg/ml heparin (all from Sigma). The number of floating spherical colonies (neurospheres) was counted after 6 days when the spheres possessed a diameter >0.08 mm. Total numbers of neurosphere-forming NSCs in the adult brain were calculated from the following: volume in which SEZ cells were suspended (usually 600 μl), volume applied in the culture, and number of resultant neurospheres in the culture.

### Corticosterone Assay

Blood was collected at the end of separation on P14 for the early-MSD group and on P21 for the early-, late-, and extended-MSD groups. The blood was allowed to clot undisturbed for 1 h and was centrifuged at 2,000 × *g* for 15 min. The corticosterone levels were assayed by ELISA (Cayman Chemical, United States) according to the manufacturer’s protocol with slight modification at a dilution of 1:1000.

### 5-Ethynyl-2′-Deoxyuridine Short-Term and 5-Bromo-2′-Deoxyuridine Long-Term Labeling and Immunohistochemistry

Immediately after the last MSD at P14, the mice were injected with a single dose of 5-ethynyl-2′-deoxyuridine (EdU) (Life Technologies; 50 mg/kg, i.p.) and sacrificed after 2 h. After deep anesthesia with 60 mg/kg ketamine and 12 mg/kg xylazine in PBS, the mice were transcardially perfused with 4% paraformaldehyde solution. Their brains were removed and post-fixed with the same fixative overnight, cryoprotected with 20% sucrose in PBS at 4°C, and then cryosectioned coronally at a thickness of 20 μm using a Leica cryostat (CM3050S). The sections were permeabilized with 0.3% Triton X-100 for 5 min followed by Click-iT EdU labeling using Alexa Fluor-555 azide (Invitrogen, United States) for 30 min at room temperature according to the manufacturer’s protocol. Then, after extensive washing with PBS containing 0.05% Tween-20 (PBS-T), the sections were incubated with Hoechst 33258 (1 μg/ml; Sigma) for 30 min.

P15 mice were injected with 5-bromo-2′-deoxyuridine (BrdU) (Sigma; 50 mg/kg, i.p.) every 3 h for a total of five injections and sacrificed 6 weeks after the last injection. After deep anesthesia, the mice were transcardially perfused with 4% paraformaldehyde solution. Brains were removed and post-fixed with the same fixative overnight, cryoprotected with 20% sucrose in PBS at 4°C, and then cryosectioned coronally at a thickness of 20 μm using a Leica cryostat (CM3050S). The sections were irradiated in a microwave for 5 min in 10 mM citrate buffer (pH 6.0) and heated to over 90°C, followed by permeabilization with 0.3% Triton-X-100 for 5 min and then blocking with 10% normal goat serum in PBS-T for 1 h. The primary anti-BrdU rat monoclonal antibody (SC-56258, Santa Cruz Biotechnology), used at a 1:200 dilution alone, with an anti-NeuN mouse monoclonal antibody (Clone A60, Millipore) at a 1:500 dilution or with anti-GFAP mouse monoclonal antibody at a 1:1000 (Clone GA5, Millipore) and anti-S100β rabbit polyclonal antibody at 1:500 (ab41548, Abcam), was applied to sections for overnight incubation at 4°C. Then, after extensive washing with PBS-T, the sections were incubated with Alexa Fluor^®^-conjugated secondary antibodies (Invitrogen, United States) at a dilution of 1:4000 overnight at 4°C, followed by Hoechst 33258 (1 μg/ml; Sigma) for 30 min.

The estimation of total numbers of EdU^+^ cells in the SEZ and SGZ or BrdU^+^ cells in the SEZ, DG, and BLA was determined using the optical dissector method (Coggeshall and Lekan, 1996) in every eighth section for the whole brain. Fluorescence images were captured using an Olympus microscope (BX51) equipped with an Olympus DP73 digital camera and a Leica laser scanning confocal microscope (TCS SP8).

### Behavioral Tests

Male C57BL/6J mice were used for all behavior tests. The open-field (OF) test was conducted according to manufacturer’s protocol (O’Hara & Co., Ltd., Tokyo, Japan). The OF apparatus is a 40 cm-square gray arena enclosed by 30 cm-high walls which are housed in a soundproof chamber and the center of the floor is illuminated at 70 lux. Ten-week-old male mice were individually placed in the center of the arena and allowed to explore freely for 10 min. Their behavior was recorded using a video tracking system and infrared photobeam sensors. The horizontal activity (total distance), vertical activity (number of rearing behaviors measured by counting the number of photobeam interruptions), and time spent in the central region comprising 36% of the total area were analyzed using tracking software (TimeOFCR1, O’Hara & Co., Ltd., Tokyo, Japan). The total distance was used as an index of locomotor activity in a novel environment, rearing numbers were used to assess stereotypic behavior, and the percentage of time spent in the central area was used as an index of anxiety.

The contextual and cued fear conditioning was measured using an O’Hara & Co., Ltd., Tokyo, Japan, apparatus. Conditioned training and testing sessions were conducted during the daytime in a soundproof behavioral room. Conditioned fear memory was evaluated by contextual and cued tests. The contextual test was used as a hippocampus-dependent learning task, while the cued test was used as a hippocampus-independent learning task. The chamber used for the conditioning training and contextual test was a rectangular clear Plexiglas chamber (width × depth × height: 34 × 26 × 29 cm, 200 lux). The chamber floor consisted of 46 stainless steel rods with a diameter of 2 mm, placed 4 mm apart. A shock generator was connected to the rods via a cable harness. The cued test chamber was a white opaque triangular chamber (35 × 35 × 40 cm, 100 lux). Both chambers were placed in a soundproof box facing a ceiling-mounted CCD camera connected to a video monitor. The training (conditioning) session consisted of three tone-shock pairings. Sixteen-week-old mice were placed into the bright rectangular chamber and allowed to explore freely for 2 min. A tone stimulus (auditory cue, 60 dB white noise) was presented as the conditioned stimulus (CS) for 30 s, followed by a mild foot shock (aversive stimulus, 2 s, 0.5 mA) as the unconditioned stimulus (US). Two more CS–US pairings were given at 2-min intervals. The contextual and cued fear conditioning test was conducted at 1 and 21 days after the training to assess recent and remote fear memory, respectively. In the contextual test, the mice were replaced into the same context as the conditioning chamber for 5 min without receiving the foot shock and auditory cue. One hour after the context testing, the cued test was conducted in an altered context. Mice were placed in a dark triangular chamber for 5 min (altered-contextual phase) and then the auditory cue was turned on for 3 min (cued phase). One day after the remote memory testing, extinction training was performed. Mice were exposed to the same procedure as in the training session but without shocks. The extinction training was repeated on three subsequent days. One day after the last extinction training, the contextual/cued test was employed to assess extinction. Freezing behavior was defined as a lack of movement for >2 s, which was measured by video tracking software (O’Hara & Co., Ltd., Tokyo, Japan). The percentage of time spent freezing was used as an index of fear memory during all sessions.

Spatial memory was assessed using the Barnes maze test (O’Hara & Co., Ltd., Tokyo, Japan) according to the manufacturer’s protocol. On a white circular platform with a diameter of 1 m, 16 holes were equally distributed around the perimeter, one of which was equipped with an escape box. The Barnes maze test was begun at 16 week old in place of the fear conditioning test. After habituation to the apparatus, each mouse was placed at the center and, over a period of 5 min, the time and number of errors required to enter the escape box was measured. Three trials per day were conducted for eight consecutive days. On day 9, a 3-min probe trial was conducted without the escape box to confirm that the spatial memory of the target was acquired based on navigation by distal environmental room cues. As for the reversal task, the target escape box was moved to a new position opposite the original one. After the training trials for another eight consecutive days, the mice received a probe trial.

### Statistical Analysis

Data were analyzed using Student’s *t*-test or analysis of variance (ANOVA). Statistical analysis was performed using GraphPad Prism 6 software for Macintosh (GraphPad Software, United States). Comparisons between the data from two groups were analyzed using an unpaired *t*-test. Multiple group comparisons were made with one-way or repeated-measured analysis of variance (ANOVA) followed by *post hoc* Tukey’s test when significant main effects were detected. The normal distribution of the data was checked by Shapiro–Wilk test and if the normality failed, non-parametric Mann–Whitney *U* test or Kruskal–Wallis test followed by *post hoc* Dunn’s multiple comparisons test was used. All data are presented as the mean ± SEM and a *p-*value of <0.05 was considered statistically significant.

## Results

### Early Life Stress Alters Neural Stem Cell Population Size

**FIGURE 1:**
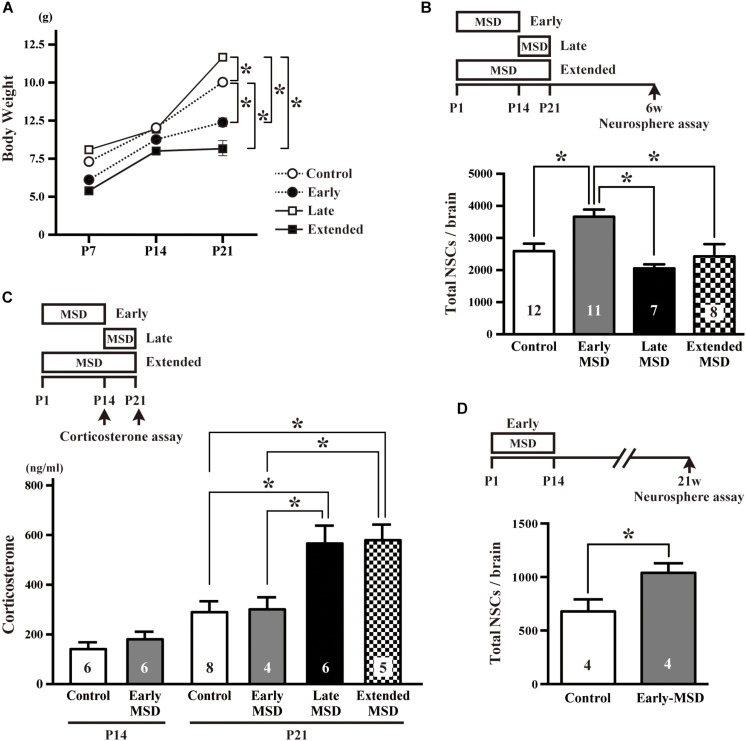
Effects of early life stress on the number of neural stem cells (NSCs) and corticosterone levels. **(A)** The pup’s bodyweight at P7, P14, and P21. **(B)** The total number of NSCs was determined by a colony-forming neurosphere assay at 6 weeks of age after maternal and social deprivation (MSD) between postnatal day P1–P14 (early), P15–P21 (late), or P1–P21 (extended). **(C)** Corticosterone levels were measured at P14 for early-MSD or at P21 for early-, late-, and extended-MSD. **(D)** The total number of neurosphere-forming NSCs was determined at 21 weeks of age. Data represent the mean ± SEM and *n* values are shown in the columns. *p < 0.05 by two-way repeated measures ANOVA **(A)**, one-way ANOVA **(B,C)**, followed by Tukey’s *post hoc* comparison or by Student’s *t*-test.

We first isolated each pup from its dam and littermates between P1–P14 (early-MSD), P15–P21 (late-MSD), or P1–P21 (extended-MSD) and measured bodyweight of the pups at P7, P14, and P21 to evaluate the effect of MSD on their growth rate. While the bodyweight was comparable among groups until P14, it was significantly higher in the late-MSD (11.71 ± 0.22 g) and significantly lower in the early- (7.34 ± 0.28 g) and extended-MSD (5.70 ± 0.50 g) compared to control group (10.04 ± 0.43 g) (Figure 1A, two-way repeated-measures ANOVA; group effect, *F*_(__3_,_73__)_ = 20.49, *p* < 0.0001; time effect, *F*_(__2_,_146__)_ = 315.2, *p* < 0.0001, *post hoc* Tukey’s multiple comparisons test). However, the early-MSD pups caught up with the control pups until 6 weeks old (early-MSD, 35.58 ± 1.33 g vs. control, 35.48 ± 1.64 g). Then, we sought to determine whether or not the size of the NSC population in the adult brain was altered by early life stress, which may show dissimilar effects depending on differences in the timing and duration of the separation episodes. At 6 weeks of age, we performed a colony-forming neurosphere assay in a low cell-density culture condition, in which single NSCs clonally generate neurospheres (Coles-Takabe et al., 2008; Higashi et al., 2008). We found that the total number of neurosphere-forming NSCs in the SEZ was significantly higher in the early-MSD mice (3666.4 ± 223.3, mean ± SEM) compared to controls (2589.6 ± 235.7), late-MSD (2053.7 ± 125.0), or extended-MSD (2432.3 ± 378.4) mice (Figure 1B; one-way ANOVA, *F*_(__3_,_33__)_ = 6.818, *p* = 0.0011).

We speculated that the HPA axis response to the stress was activated in late- and extended-MSD mice after P14. Indeed, while the corticosterone levels were comparable at P14 between the early-MSD and control mice (Figure 1C left, *t*_(__10__)_ = 0.9476, *p* = 0.3657), the levels after the last stress significantly increased in the late- and extended-MSD mice compared to controls (Figure 1C right; one-way ANOVA: *F*_(__3_,_19__)_ = 7.483, *p* = 0.0017; *post hoc* Tukey’s multiple comparisons test: control vs. late-MSD, *p* = 0.0086; control vs. extended-MSD, *p* = 0.0096), suggesting that high corticosterone levels elicited by stress after P14 in late- and extended-MSD abrogated the increase of NSC population size observed in early-MSD. On the other hand, early MSD showed comparable levels of the corticosterone to controls at P21 (Figure 1C right; *post hoc* Tukey’s test: control vs. early-MSD, *p* = 0.9991), suggesting that the stress during the SHRP did not activate the HPA-axis and, therefore, that early- and late-MSD impose different long-term impacts on the neural development. We further investigated whether or not the effects of early-MSD on NSC population size persist long into adulthood by means of the neurosphere assay. We found that the total number of NSCs in the SEZ remained higher at 21 weeks in mice from the early-MSD group than the control group, after the completion of behavioral tasks described below (Figure 1D; *t*_(__6__)_ = 2.504, *p* = 0.0463).

**FIGURE 2:**
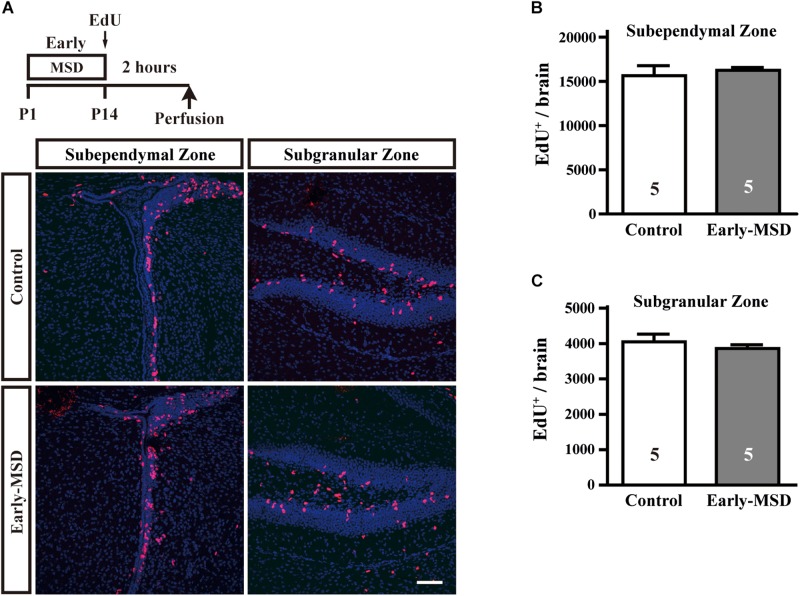
Effects of early life stress on the proliferation of neural precursor cells. **(A)** Mice received a single dose of EdU at P14 and sacrificed 2 h after the injection. Incorporation of EdU was visualized on the coronal cryosections. **(B,C)** The total numbers of EdU^+^ cells were counted in the SEZ **(B)** and SGZ **(C)**. Data represent the mean ± SEM and *n*-values are shown in the columns. Scale bars: 100 μm.

Next, we examined numbers of proliferating cells immediately after early-MSD by injecting EdU and labeling dividing cells for 2 h (Figure 2A). We found that the total number of EdU^+^ cells were comparable between early-MSD and control groups (Figures 2B,C; *t*_(__8__)_ = 0.5056, *p* = 0.6286 for SEZ; *t*_(__8__)_ = 0.7786, *p* = 0.4586 for SGZ). Most of the labeled cells are considered vigorously dividing transit amplifying cells (TACs), progeny of NSCs, because NSCs become relatively quiescent and have much longer cell cycle times than TACs. Therefore, it is possible that the expansion of NSC population was not reflected to the number of EdU^+^ cells at this point. Additionally, no EdU^+^ cells were detected in other brain regions, including the BLA in both groups.

**FIGURE 3:**
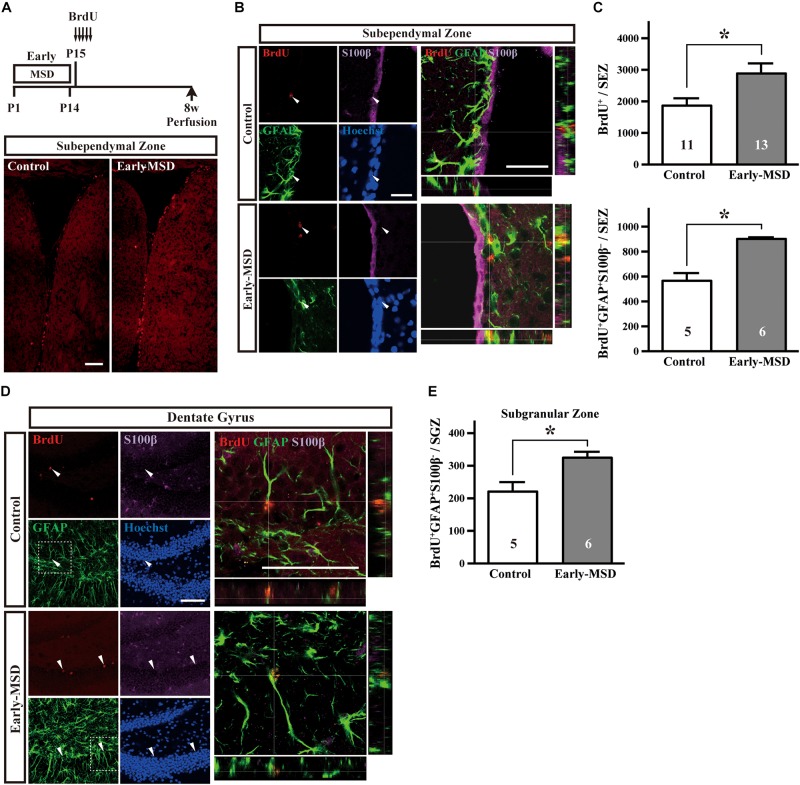
Effects of early life stress on the number of neural stem cells (NSCs) in the adult brain. **(A)** Mice received five injections of BrdU at P15 with a 3-h interval and were subjected to immunohistochemical analysis at 8 weeks of age. Coronal cryosections were immunostained for BrdU. **(B)** Coronal cryosections were immunostained for BrdU, GFAP, and S100β and the merged pictures with orthogonal views of confocal *z*-stack images are shown at the right. Arrowheads indicate BrdU^+^ GFAP^+^ S100β^–^ cells in the subependymal zone (SEZ). **(C)** The total numbers of BrdU^+^ (top) and BrdU^+^ GFAP^+^ S100β^–^ (bottom) cells in the SEZ were counted. **(D)** Coronal cryosections were immunostained for BrdU, GFAP, and S100β. The dotted box areas are enlarged and the merged pictures with orthogonal views of confocal *z*-stack images are shown at the right. Arrowheads indicate BrdU^+^ GFAP^+^ S100β^–^ cells in the subgranular zone (SGZ). **(E)** The total numbers of BrdU^+^ GFAP^+^ S100β^–^ cells in the SGZ were counted. Data represent the mean ± SEM and *n*-values are shown in the columns. *p < 0.05 by Student’s *t*-test. Scale bars: 100 μm **(A)** or 50 μm **(B,D)**.

The NSC population was assessed by long-term BrdU incorporation: BrdU was administered on P15 and BrdU-positive cells in the SEZ were assessed at 8 weeks of age. We used BrdU, instead of EdU, because BrdU seems less cytotoxic than EdU for long-term stem cell labeling (Neef and Luedtke, 2011). During this period, vigorously dividing TACs either dilute BrdU by further divisions until levels become undetectable, migrate from the SEZ to the olfactory bulb, or die. Therefore, BrdU^+^ cells that remain in the SEZ at 8 weeks largely consist of NSCs. Results showed that early-MSD significantly increased the number of BrdU^+^ cells in the whole SEZ compared to controls (Figures 3A,B top; *t*_(__22__)_ = 2.483, *p* = 0.0211). These results were further confirmed by triple immunostaining for BrdU, GFAP, and S100β because NSCs are GFAP^+^ S100β^–^ whereas astrocytes are GFAP^+^ S100β^+^ (Raponi et al., 2007). We observed the significant increase of the number of BrdU^+^ GFAP^+^ S100β^–^ cells in the early-MSD mice compared to controls (Figures 3B,C bottom; *t*_(__9__)_ = 5.755, *p* = 0.0003). Neural precursor cells are also present in the SGZ of the dentate gyrus, which migrate radially to the granular cell layer and differentiate to express neuronal markers. Quiescent NSCs that have incorporated BrdU at P15 should remain in the SGZ and retain the label at 8-week-old and they can be identified as GFAP^+^ S100β^–^ radial glia-like cells (Kempermann et al., 2004; Raponi et al., 2007; Bonaguidi et al., 2011). We revealed that the total number of BrdU^+^ GFAP^+^ S100β^–^ cells in the SGZ was significantly higher in the early-MSD than the control (Figures 3D,E; *t*_(__9__)_ = 3.065, *p* = 0.0135). Thus, early life stress during the SHRP increases the size of the NSC population both in the SEZ and SGZ.

### Early-MSD Enhances Neurogenesis

**FIGURE 4:**
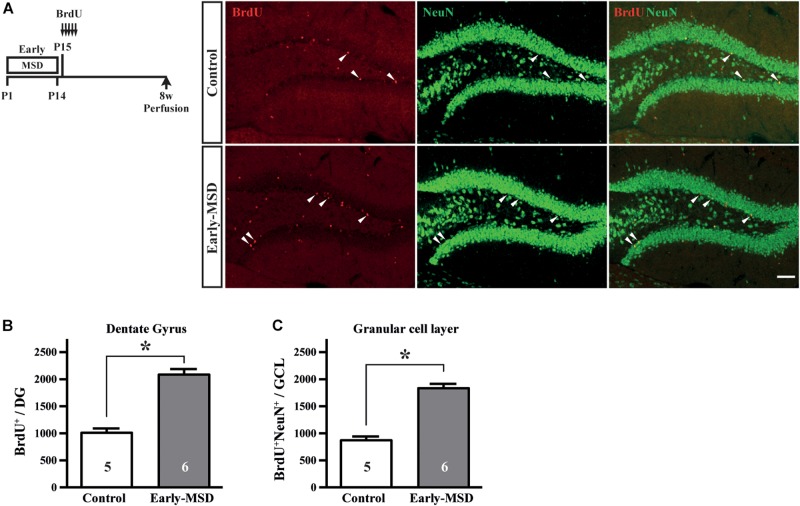
Effects of early life stress on neurogenesis in the dentate gyrus. **(A)** Mice received five injections of BrdU at P15 with a 3-h interval and were subjected to immunohistochemical analysis at 8 weeks of age. The same mice presented in Figure 3C bottom, E were analyzed. Coronal cryosections were immunostained for BrdU and NeuN and confocal images are shown. Arrowheads indicate BrdU^+^ cells that co-expressed NeuN. **(B)** The total numbers of BrdU^+^ cells in the whole dentate gyrus were counted. **(C)** The total numbers of BrdU^+^ NeuN^+^ cells in the granular cell layer were counted. Data represent the mean ± SEM and *n-*values are shown in the columns. *p < 0.05 by Student’s *t*-test. Scale bars: 100 μm.

**FIGURE 5:**
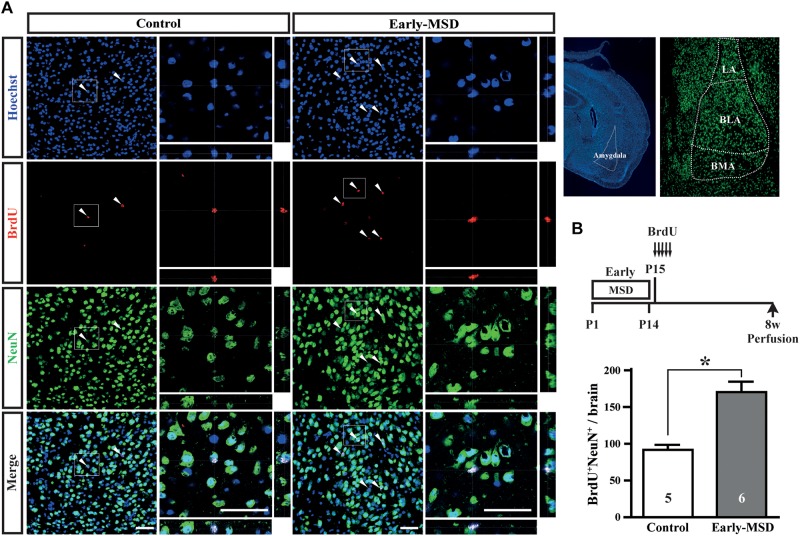
Effects of early life stress on neurogenesis in the amygdala. **(A)** Mice received five injections of BrdU at P15 with a 3-h interval and were subjected to immunohistochemical analysis at 8 weeks of age. The same mice presented in the Figure 3C bottom, E were analyzed. The structures of the amygdala and its subnuclei in the adult brain are shown to the right. Coronal cryosections were immunostained for BrdU and NeuN and confocal images are shown. Boxed areas were enlarged and shown as orthogonal views of confocal *z*-stack images. **(B)** Double positive cells for BrdU and NeuN were counted. Data represent the mean ± SEM and *n-*values are shown in the columns. *p < 0.05 by Student’s *t*-test. Scale bars: 50 μm. LA, lateral amygdala; BLA, basolateral amygdala; BMA, basomedial amygdala.

We next examined if the expanded NSC population after early-MSD results in enhanced neurogenesis. Neural precursor cells that divide and incorporate BrdU at P15 in the SGZ radially migrate, differentiate into granular cells that co-expressed a neuronal marker NeuN, and survive until 8 weeks in the dentate gyrus (Figure 4A). More BrdU^+^ cells in the whole dentate gyrus (Figure 4B; *t*_(__9__)_ = 7.950, *p* < 0.0001) and more BrdU^+^ NeuN^+^ cells in the granular cell layer (Figure 4C; *t*_(__9__)_ = 8.851, *p* < 0.0001) were observed in the hippocampus of mice after early-MSD than control. Because EdU incorporation into the SGZ cells immediately after the last separation of early-MSD was comparable to the control (Figure 2), it is possible that the differentiation of neural precursors or the survival of differentiated neurons was enhanced by early-MSD. Interestingly, we noticed that BrdU-positive cells were also present in the amygdala, although this region is not considered a classic neurogenic region in the adult brain. BrdU-positive cells observed in the basolateral amygdala (BLA), at least in part, co-expressed the neuronal marker NeuN, and a significantly higher number of BrdU^+^ NeuN^+^ cells were observed after early-MSD (Figures 5A,B; *t*_(__9__)_ = 4.680, *p* = 0.0012). Since no EdU^+^ cells were detected in the amygdala at P14, those BrdU^+^ NeuN^+^ cells may have incorporated BrdU somewhere outside the amygdala and migrate to it. However, our data do not exclude a possibility that our 2-h labeling was not sufficient to label the neurogenesis at P14 mouse amygdala. Indeed, *in situ* neurogenesis in the adult amygdala has been reported (Jhaveri et al., 2018). Although the migratory pathway from the ventricular zone of the embryonic brain to the amygdala is reported (Carney et al., 2006), there is no evidence of those cell migration from the SEZ or other areas to the amygdala postnatally, which remains to be investigated. These results suggest that the expansion of the neural precursor population after early-MSD results in enhanced neurogenesis in the dentate gyrus and amygdala.

### Behavioral Changes After Early Life Stress

**FIGURE 6:**
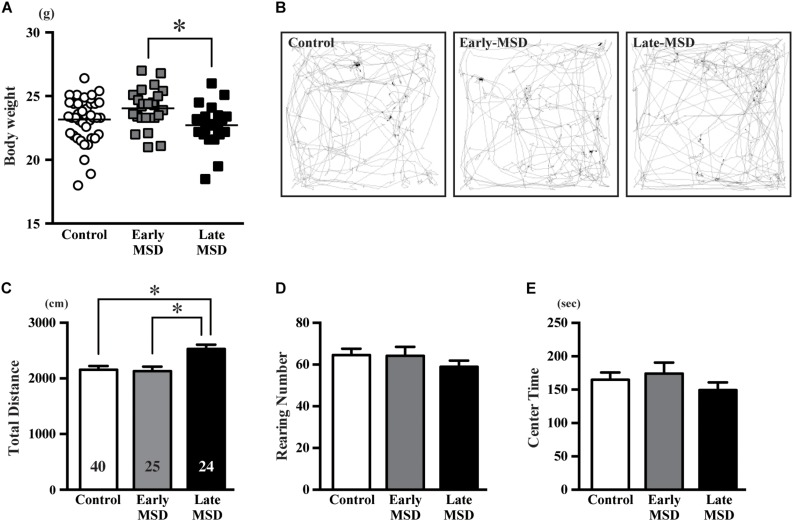
The open-field test. **(A)** The bodyweight of each mouse at 10 weeks of age is shown. Horizontal bars indicate the means. **(B**–**E)** Ten-week-old male mice exposed to early- or late-MSD and control mice were subjected to the open-field test. **(B)** Representative trajectories of the control, early-MSD, and late-MSD mice in the open-field chamber during the 10-min test period are shown. **(C**–**E)** Total traveled distance **(C)**, number of rearing behaviors **(D)**, or time spent in the center area **(E)** were compared among the control, early-MSD, and late-MSD mice. Data represent the mean ± SEM and *n-*values are shown in the columns. ^∗^*p* < 0.05 by one-way ANOVA followed by Tukey’s *post hoc* comparison.

Altered neurogenesis is associated with mood and emotional changes, which can be detected by behavioral batteries in rodents. We first measured the body weight of each mouse, as a 3-h separation from the dam or high corticosterone levels could result in growth retardation. Indeed, the body weights of late-MSD mice at 10 weeks of age, which showed elevated plasma corticosterone levels after the stress, were lower than those of the early-MSD mice (Figure 6A; one-way ANOVA: *F*_(__2_,_86__)_ = 4.137, *p* = 0.0193; *post hoc* Tukey’s test: early-MSD vs. late-MSD, *p* = 0.0170). However, neither group showed significant differences compared to controls; therefore, we concluded that the growth retardation, if any, would not significantly affect behavioral analyses. First, we conducted an OF test, in which we evaluated the total traveled distance, the number of rearing behaviors and time spent in the center area (Figure 6B). The late-MSD mice displayed hyperactivity and enhanced locomotion compared to control and early-MSD mice as demonstrated by a significant increase in the total distance traveled in the OF test (Figure 6C; one-way ANOVA: *F*_(__2_,_86__)_ = 0.7626, *p* = 0.0009; *post hoc* Tukey’s test: control vs. late-MSD, *p* = 0.0020; early-MSD vs. late-MSD, *p* = 0.0031). No significant effects on the number of rearing behaviors nor the time spent in the center area were detected (Figures 6D,E).

**FIGURE 7:**
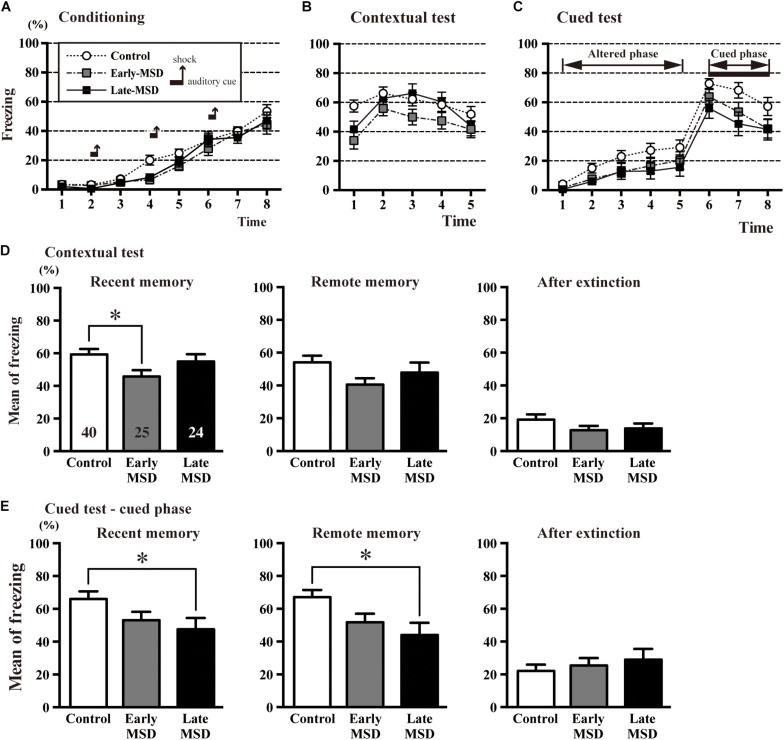
The contextual and cued conditioning test. **(A**–**C)** Durations of the % freezing time during the training session (conditioning) **(A)**, contextual test **(B)**, and cued test **(C)** are shown. In the training session, mice were introduced to the tone stimuli (horizontal bars) followed by foot shocks (vertical arrows), which resulted in a gradual increase in freezing time. **(D,E)** The mean % freezing time of the contextual test **(D)** and cued phase of the cued test **(E)** were evaluated 1 day after the training session as an indicator of recent memory (left), 21 days after the training session as an indicator of remote memory (middle), and after extinction training (right). In the recent contextual test, early-MSD mice showed a significant decrease in the mean freezing time as compared to control, while the significant decrease in the cued memory was observed in late-MSD. Data represent the mean ± SEM and *n-*values are shown in the columns. ^∗^*p* < 0.05 by one-way ANOVA followed by Tukey’s *post hoc* comparison **(D)** or Kruskal–Wallis test followed by Dunn’s multiple comparisons test **(E)**.

Next, we examined associative fear learning between environmental context or auditory cues and aversive experiences using the conditioning test to determine the effect of MSD on fear memory. During the training (conditioning) session, the mice exhibited reliable fear conditioning as measured by an increase in freezing time throughout the conditioning (Figure 7A). Freezing time duration was comparable among the groups (two-way repeated measures ANOVA, group effect: *F*_(__2_,_86__)_ = 2.314, *p* = 0.1050). One day after the training, recent fear memory was analyzed by contextual and cued tests (Figures 7B,C). In the contextual test, early-MSD mice showed a significantly decreased mean freezing time compared with controls (Figure 7D left; one-way ANOVA: *F*_(__2_,_86__)_ = 3.196, *p* = 0.0458; *post hoc* Tukey’s test: control vs. early-MSD, *p* = 0.0357). To assess if this contextual fear memory persisted for a long period, we investigated contextual remote fear memory, which was examined 21 days after the conditioning. We found a non-significant but similar decrease in the mean freezing time in early-MSD mice (Figure 7D middle; one-way ANOVA: *F*_(__2_,_86__)_ = 2.229, *p* = 0.1138). The cued test consisted of two phases: an altered phase in a novel chamber but without the auditory cue, and a cued phase with the cue. The test was performed 1 h after the contextual test for both recent and remote memory analyses. The late-MSD mice exhibited a significant decrease in the mean freezing time not only for the recent but also for the remote memory compared to control mice (Figure 7E left and middle; Kruskal–Wallis test: *p* = 0.0278; *post hoc* Dunn’s multiple comparisons test. control vs. late-MSD, *p* = 0.0432 for recent memory; Kruskal–Wallis test: *p* = 0.0188, Dunn’s multiple comparisons test, control vs. late-MSD, *p* = 0.0313 for remote memory). The early-MSD mice showed a non-significant but similar decrease in the mean freezing time for recent and remote memory. After the remote memory test, mice were exposed to the same conditions as the training sessions but without electrical shocks and were tested for contextual and cued memory. After the extinction of fear memory, the mean freezing times became lower in all groups with no significant differences between them (Figures 7D,E right).

**FIGURE 8:**
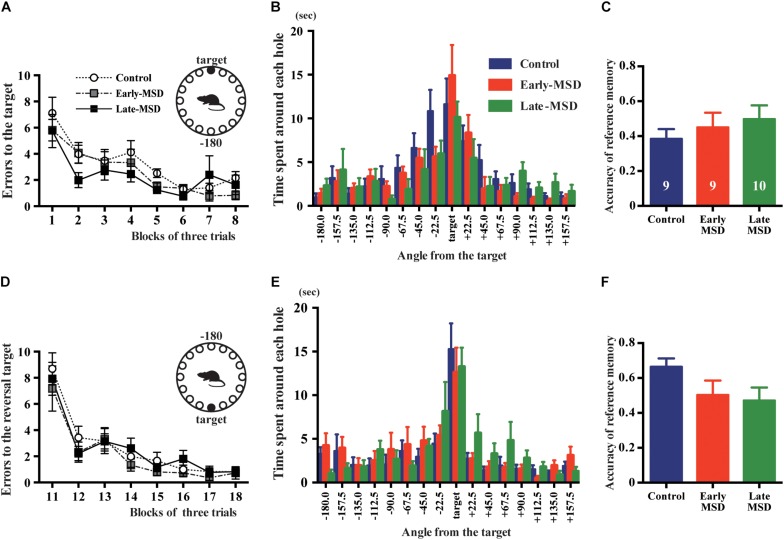
Spatial memory test. Early- and late-MSD and control mice were assessed for the spatial memory by Barnes maze test. **(A)** Errors in entering the target escape box were measured during the training. **(B)** In the probe test, time spent around each hole was measured in relation to the angle of the hole to the target. **(C)** The accuracy of the reference memory was scored as the ratio of time spent around the target divided by the time spent around the target and neighboring holes. **(D)** After the reversal of the target location, errors in entering the target escape box were measured during the training. **(E)** In the reversal probe test, time spent around each hole was measured in relation to the angle of the hole to the target. **(F)** The accuracy of the reference memory was scored as described above. Data represent the mean ± SEM and *n-*values are shown in the columns.

The formation and maintenance of contextual and cued fear memory are largely mediated by the function of the hippocampus and amygdala (Phillips and LeDoux, 1992; LeDoux, 2000). To discriminate the involvement of these regions, we tested spatial memory, which is hippocampus-dependent, using the Barnes maze test. All groups of mice learned to locate the target hole during the training period, as indicated by gradual reductions in the number of search errors (Figure 8A; *F*_(__2,__25__)_ = 1.545, *p* = 0.2330). In the probe test, we could not detect any significant differences regarding the time spent around the target hole (*F*_(__2,__25__)_ = 0.5784, *p* = 0.5681) nor accuracy of the reference memory (*F*_(__2,__25__)_ = 0.5953, *p* = 0.5681) among the groups (Figures 8B,C). In addition, we conducted a reversal test, in which the target was moved to the opposite position from the original target, and found no significant differences in the number of errors to the reversal target (*F*_(__2,__25__)_ = 0.5451, *p* = 0.5865) nor in the time spent around the target hole (*F*_(__2,__25__)_ = 0.1619, *p* = 0.8514) nor accuracy of the reference memory (*F*_(__2,__25__)_ = 2.140, *p* = 0.1387) in the probe test (Figures 8D–F). These results suggest that hippocampus-mediated spatial memory was not affected by early life stress.

## Discussion

In the present study, we show for the first time that early life stress exhibits distinct effects on the activity of the NSC-neurogenesis system in the adult brain, depending on the timing and duration of the stress. MSD during the SHRP increases the size of the NSC population compared to controls; however, the same stress extended beyond the SHRP has the opposite effects. We presented two lines of evidence for the expansion of the NSC population after early-MSD: the estimated number of total NSCs using the neurosphere assay and the BrdU label retention assay in the SEZ. In the DG of the hippocampus, the estimation of NSCs pool was analyzed by BrdU long-term labeling co-expressed with GFAP and negative for S100β in the SGZ at 8 weeks. There have been very few studies to date evaluating NSC population size after early life events; however, our results are consistent with data obtained in another rodent study (Kippin et al., 2004). During the early postnatal period, the number of neurosphere-forming NSCs decreases from its peak around birth to relatively stable levels at 6 weeks and beyond (Hitoshi et al., 2004). Therefore, it is possible that early-MSD augments factor(s) that enhance survival or prevent the differentiation of NSCs, although the identity of the factor(s) remains to be determined. Our data do not rule out a possibility that MSD alters maternal behavior and mother–pup attachment, which is essential for healthy neurodevelopment of the pups. Indeed, maternal nursing behaviors are reported to be more active in the dams, which experience MSD (Zimmerberg and Sageser, 2011). However, we do not think the enhanced maternal care greatly contributed to the expansion of NSC population size in the early-MSD because early-MSD and littermate control pups were bred in the same dam.

We also demonstrated an increase in neurogenesis in the BLA in addition to the dentate gyrus of the hippocampus, a classical neurogenic region, after early-MSD. Although amygdalar development is mostly complete by E15.5 in mice (Soma et al., 2009), neurogenesis in the BLA of the adult brain has been reported (Jhaveri et al., 2018), which could modify the function of the amygdala. Notably, the BLA is the main area for the acquisition and consolidation of fear memory, and their activities are thought to support hippocampal long-term potentiation and enhance hippocampal output to other brain regions (Paz et al., 2006; Bergado et al., 2007). It is reported that amygdalar plasticity is under the influence of stress, leading to behavioral changes, such as the manifestation of anxiety (Mitra et al., 2009). Our data may provide an explanation to this notion that postnatal neurogenesis could alter the function and plasticity of the amygdala. However, little was known about external stimuli and environmental cues that alter the production of new neurons in the postnatal amygdala, and this is the first report showing that early life stress during the SHRP enhances amygdalar neurogenesis. Interestingly, early life stress leads to persistent alterations in amygdalar circuitry and function in mice and humans (Cohen et al., 2013). Based on our data showing that mice exposed to early-MSD respond less to amygdala-mediated cued fear memory than control mice, it would be intriguing to investigate whether or not new neurons labeled by BrdU at P15 contribute to this neural network and modify the function of the amygdala. It also remains to be determined whether or not the amygdalar neurogenesis and function are modified by the late-MSD, which resulted in the significantly reduced amygdala-mediated cued fear memory.

Currently, enhanced adult neurogenesis is thought to facilitate the clearance of fear memory and improve coping and recovery from psychological damage caused by stress, which could explain the resilience to stress (Franklin et al., 2012; Pfau and Russo, 2015; Anacker et al., 2018). Repeated early life stress elicits a predictive adaptive response where past stressful experiences augment the ability to cope with future stress (Gluckman et al., 2007). The match/mismatch hypothesis of psychotic diseases states that early life stress could have adaptive value to individuals facing later stressful experiences (Santarelli et al., 2014). Our data, demonstrating that early MSD increases neurogenesis in the dentate gyrus of the hippocampus and decreases the response to fear memory, are similar to results from mice after chronic treatment with memantine, a non-competitive inhibitor of the *N*-methyl-D-aspartate type glutamate receptor (Ishikawa et al., 2016). Similar results showing reduced contextual fear memory have also been reported in runner mice (Akers et al., 2014; Gao et al., 2018). Thus, the decreased response to recent contextual fear memory after early-MSD could be related to the increase in the hippocampal and amygdalar neurogenesis.

## Dedication

This article is dedicated to Dr. Kazuhiro Ikenaka who passed away during the preparation of this manuscript.

## Data Availability Statement

All datasets generated for this study are included in the article/supplementary material.

## Ethics Statement

The animal study was reviewed and approved by the Animal Care and Use Committee of the Shiga University of Medical Science.

## Author Contributions

NK and SH designed the research. KD, TF, NK, NM, and SH performed the research. KD, NK, and SH analyzed the data and prepared the manuscript. KI and SH supervised the research.

## Conflict of Interest

The authors declare that the research was conducted in the absence of any commercial or financial relationships that could be construed as a potential conflict of interest.
